# Integrating innovative sensors

**Published:** 2012-06-15

**Authors:** Jennifer Caffarel, Axel Saalbach, Alberto Bonomi, Joerg Habetha, Matthew Harris, Patrik Schauerte, Juan Perez Villacastin, Ramon Bover, Christian Zugck, Juergen Vogt, Arcadi García Alberola, John Cleland

**Affiliations:** Philips Research Eindhoven, The Netherlands; Philips Research Hamburg, Germany; Philips Research Eindhoven, The Netherlands; Philips Research Eindhoven, The Netherlands; Philips Research Eindhoven, The Netherlands; Innere Medizin, Kardiologie, Universitätsklinikum Aachen, Germany; Cardiologia, Instituto Cardiovascular, Hospital Clínico San Carlos, Madrid, Spain; Cardiologia, Instituto Cardiovascular, Hospital Clínico San Carlos, Madrid, Spain; Innere Medizin III, Kardiologie, Angiologie und Pulmonologie, Universitätsklinikum Heidelberg, Germany; Herz- und Diabeteszentrum NRW, Bad Oeynhausen, Germany; Servicio de Cardiología, Hospital Universitario Virgen de la Arrixaca, Murcia, Spain; Cardiology, Castle Hill Hospital, Hull, UK

**Keywords:** telemonitoring, heart failure, decompensation

## Abstract

**Introduction:**

Heart failure is a significant and growing health problem. To enable a preventative health care system in heart failure, a move is required from the current, intermittent episodical treatment to continuous and ubiquitous access to medical excellence. Home telemonitoring systems that monitor vital body signs and symptoms with wearable technology, have been proposed to enhance treatment and anticipation of adverse events in heart failure patients (decompensation).

**Aims and objectives:**

We investigated whether the data recorded in a home telemonitoring system using innovative sensors had predictive value in detecting heart failure related events.

**Methods:**

In the MyHeart observational clinical trial, 148 heart failure patients were followed daily for up to one year using an innovative telemonitoring system recording symptoms questionnaires, basic parameters such as body weight and blood pressure, as well as advanced information provided by a bed sensor monitoring night heart rate and activity and a thorax bioimpedance (BIM) device, designed to monitor fluid accumulation in the lungs. The area under the receiver operator characteristic curve (AUC) was used to evaluate the predictive value for heart failure related events of single and combined telemonitoring measurements.

**Results:**

The telemonitoring system including innovative sensors was rated positively by the patients, which is further supported by the reasonable compliance rates (>65%) measured. The most predictive variables 14 days preceding an event were thoracic bioimpedance (area under the curve of 0.70), night breathing rate and some symptoms. The initial multiparametric analysis achieved a mean accuracy of 80.6% but would require more data to confirm its generalisation ability.

**Conclusions:**

The measurements performed during the MyHeart trial with 148 patients and 12 months’ follow-up provided evidence supporting the development of predictive algorithms of decompensation to support heart failure management at home. An innovative wearable monitor of thoracic congestion by means of bioimpedance was tested and showed the highest predictive value for heart failure decompensation among a large set of monitored vital signs and symptoms.

